# LaTe_1.82(1)_: modulated crystal structure and chemical bonding of a chalcogen-deficient rare earth metal polytelluride^1^

**DOI:** 10.1107/S2053229620005094

**Published:** 2020-05-06

**Authors:** Hagen Poddig, Kati Finzel, Thomas Doert

**Affiliations:** a Inorganic Chemistry, Technische Universität Dresden, Bergstrasse 66, Dresden 01069, Germany; b Theoretical Chemistry, Technische Universität Dresden, Bergstrasse 66c, Dresden 01069, Germany

**Keywords:** modulated structure, rare earth metal polytellurides, bonding analysis, crystal structure, electrical properties

## Abstract

Crystals of the rare earth metal polytelluride LaTe_1.82(1)_, namely, lanthanum telluride (1/1.8), have been grown by molten alkali halide flux reactions and vapour-assisted crystallization with iodine. The two-dimensionally incommensurately modulated crystal structure has been investigated by X-ray diffraction experiments. In contrast to the tetra­gonal average structure with unit-cell dimensions of *a* = 4.4996 (5) and *c* = 9.179 (1) Å at 296 (1) K, which was solved and refined in the space group *P*4/*nmm* (No. 129), the satellite reflections are not compatible with a tetra­gonal symmetry but enforce a symmetry reduction. Possible space groups have been derived by group–subgroup relationships and by consideration of previous reports on similar rare earth metal polychalcogenide structures. Two structural models in the ortho­rhom­bic superspace group, *i.e.* *Pmmn*(α,β,)000(−α,β,)000 (No. 59.2.51.39) and *Pm*2_1_ *n*(α,β,)000(−α,β,)000 (No. 31.2.51.35), with modulation wave vectors *q*_1_ = αa* + βb* +  c* and *q*_2_ = −αa* + βb* +  c* [α = 0.272 (1) and β = 0.314 (1)], have been established and evaluated against each other. The modulation describes the distribution of defects in the planar [Te] layer, coupled to a displacive modulation due to the formation of different Te anions. The bonding situation in the planar [Te] layer and the different Te anion species have been investigated by density functional theory (DFT) methods and an electron localizability indicator (ELI-D)-based bonding analysis on three different approximants. The temperature-dependent electrical resistance revealed a semiconducting behaviour with an estimated band gap of 0.17 eV.

## Abstract 2

Small finite telluride anions are found as dominant species in the anionic layers of modulated LaTe_1.82(1)_, corresponding to its semiconducting properties.

## Introduction   

The structural chemistry of the rare earth metal chalcogenides *REX*_2–δ_ (*RE* = Y, La–Nd, Sm, Gd–Lu; *X* = S, Se or Te; 0 ≤ δ ≤ 0.2) with trivalent *RE* metals has attracted attention because of their structural variety in a quite small compositional range. The sulfides and selenides of this class of compounds have been intensively investigated, illuminating several different (super)structures due to different amounts of defects δ and the formation and arrangements of chalcogenide *X* ^2−^ and polychalcogenide *X_n_* ^2−^ anions for charge balancing. A comprehensive overview discussing these aspects can be found in Doert & Müller (2016 ▸). At the beginning of the 21st century, the structures of four rare earth metal polytellurides *RE*Te_2–δ_ (*RE* = La–Nd) (Stöwe, 2000*a* ▸,*b* ▸,*c* ▸, 2001 ▸), were thoroughly (re)investigated, revealing considerable differences to the sulfides and selenides of analogous compositions, while still maintaining the same general structural motif. This general structural motif of all binary polychalcogenides *REX*_2–δ_ of trivalent rare earth metals is closely related to the structure of ZrSSi (space group *P*4/*nmm*, No. 129; *a*_0_ ≃ 3.80 and *c*_0_ ≃ 8.00 Å) (Onken *et al.*, 1964 ▸; Klein Haneveld & Jellinek, 1964 ▸), which shows an alternating stacking of puckered [ZrS] slabs and square-planar [Si] layers along [001]. The binary rare earth metal chalcogenides *REX*_2–δ_ (*RE* = Y, La–Nd, Sm, Gd–Lu; *X* = S, Se or Te; 0 ≤ δ ≤ 0.2) comprise puckered [*REX*]^+^ slabs and planar chalcogenide layers, which can formally be stated as [*X*]^−^ (Doert & Müller, 2016 ▸). For electronic reasons, the chalcogenide layers of the stoichiometric *REX*_2_ compounds, especially the di­sulfides *RE*S_2_ and diselenides *RE*Se_2_, feature only *X*_2_^2−^ dianions, resulting in a distortion from an idealized square-planar net towards a planar herringbone pattern; for ditellurides *RE*Te_2_, the structural situation is not that uniform (Stöwe, 2000*a* ▸,*b* ▸,*c* ▸). Going to the off-stoichiometric *REX*_2–δ_ (0 < δ ≤ 0.2) compounds, vacancies in the planar chalcogenide layers are observed, together with *X* ^2−^ to maintain the overall net charge [*X*]^−^ for the layer. This structural change is obvious for the CeSe_1.9_ structure type, but can also be seen for the related, intrinsically disordered Gd_8_Se_15_-type structures (Doert *et al.*, 2012 ▸; Doert, Dashjav *et al.*, 2007 ▸). Hence, the two most important factors accounting for structural differences are the amount of vacancies in the chalcogenide layer and the ionic radii of the trivalent rare earth metal cations, as they largely determine the Coulomb repulsion between the anions in the [*X*]^−^ layers in this series. In addition, in accordance with the Zintl-like electron localization, the polysulfides *RE*S_2–δ_ and polyselenides *RE*Se_2–δ_ are semiconductors.

To distinguish between different anionic fragments in the chalcogenide layer, classical electron counting has been proven a simple but powerful way to describe these structures, as briefly explained for the CeSe_1.9_ structure type; the CeSe_1.9_ type is a 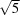 × 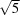 × 2 superstructure of the basic ZrSSi unit cell and crystallizes in the space group *P*4_2_/*n* (No. 86) (Plambeck-Fischer *et al.*, 1989 ▸). The planar [Se] layer of this compound is built up by four dinuclear Se_2_^2−^ anions, forming a pinwheel-like arrangement around a vacancy. The complementary isolated Se^2−^ anion is surrounded by four Se_2_^2−^ anions in a spoke-like manner (Lee & Foran, 1994 ▸). Assuming only trivalent rare earth metal cations, ten positive charges per [*REX*]^+^ layer and unit cell need to be balanced by nine atoms of the planar [*X*]^−^ layer. This is achieved by four Se_2_^2−^ anions and one isolated Se^2−^ anion. This kind of charge-ordered superstructure has only been reported once for a rare earth metal telluride, namely for CeTe_1.9_ (Ijjaali & Ibers, 2006 ▸), whereas many examples are known for the rare earth metal polysulfides and polyselenides (Doert, Graf, Lauxmann *et al.*, 2007 ▸; Grupe & Urland, 1991 ▸; Plambeck-Fischer *et al.*, 1989 ▸; Urland *et al.*, 1989 ▸; Dashjav *et al.*, 2000 ▸; Müller *et al.*, 2012 ▸).

An unusual case of charge balancing for the deficient *REX*_2–δ_ compounds has been reported for structures with a composition of *RE*Te_1.8_ (Sm, Gd–Dy) by forming larger anionic fragments (Ijjaali & Ibers, 2006 ▸; Wu *et al.*, 2002 ▸; Gulay *et al.*, 2007 ▸; Poddig *et al.*, 2018 ▸). Here, a similar enlargement of the basic lattice parameters of 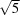 × 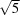 × 2 is observed, and the compounds crystallize in a 10-fold superstructure of the aristotype in *P*4/*n* (No. 85). In contrast to the respective sulfides and selenides, a motif of statistically disordered Te_2_^2−^ anions and linear Te_3_^4−^ anions are found here. Linear Te_3_^4−^ anions have rarely been reported in *REX*_2–δ_ compounds, although the presence of an Se_3_^4−^ anion was discussed for DySe_1.84_, but neglected after computational studies (van der Lee *et al.*, 1997 ▸). The bonding situation in such linear trinu­clear anions, like Te_3_^4−^ or I_3_^−^, requires the occupation of nonbonding states, similar to the situation of the prominent XeF_2_ mol­ecule. Within the concept of mol­ecular orbital (MO) theory, this situation can be described as a 3*c*–4*e* bond (Rundle, 1963 ▸; Assoud *et al.*, 2007 ▸). A density functional theory (DFT)-based study clearly evidenced such a linear Te_3_ unit in GdTe_1.8_ (Poddig *et al.*, 2018 ▸) and confirmed an alternative method of electron localization for this composition of *REX*_1.8_: ten positive charges of one puckered [*REX*]^+^ layer per unit cell are balanced by two Te_3_^4−^ anions and one Te_2_^2−^ anion.

Starting from the results of the *RE*Te_1.8_ (*RE* = Sm, Gd–Dy) compounds, we were inter­ested in identifying the structural motifs of the early rare earth metal tellurides *RE*Te_2–δ_ with δ > 0. This was especially motivated by the reported differences between the structures of LaTe_2_, CeTe_2_ and PrTe_2_ (Stöwe, 2000*a* ▸,*b* ▸,*c* ▸), and the corresponding sulfides and selenides. Structural data on tellurides *RE*Te_2–δ_ with a comparable low chalcogen content have rarely been reported; *RE*Te_1.8_ (*RE* = Sm, Gd–Dy) are a few examples (Ijjaali & Ibers, 2006 ▸; Wu *et al.*, 2002 ▸; Gulay *et al.*, 2007 ▸; Poddig *et al.*, 2018 ▸). The first results on the lanthanum compound LaTe_1.82(1)_ are presented in the following.

## Experimental   

### Synthesis   

All preparation steps were carried out in an argon-filled (5.0, Praxair) glove-box (MBraun, Garching, Germany). Crystals were grown by the addition of a small amount of I_2_ to the reaction mixture in closed silica ampoules. In a standard synthesis, 500 mg of a stoichiometric mixture of lanthanum (99.9%, Edelmetall Recycling m&k GmbH) and tellurium (Merck, >99.9%, reduced in a H_2_ stream at 673 K) were placed in a quartz tube and flame sealed under dynamic vacuum (*p* ≤ 1 × 10^−3^ mbar). The ampoule was heated slowly with a ramp of 2 K min^−1^ to 1073 K. The reaction takes place in a gradient from 1123 to 1073 K with I_2_ (Roth, >99.8%, purified by sublimating twice prior to use) as transporting agent. After 7 d, the ampoule was cooled to room temperature. As we observed a slow degrading of the compounds under atmos­pheric conditions, the samples were stored under argon.

### Powder diffraction   

Data collection was performed at 296 (1) K with an Empyrean diffractometer (PANalytical) equipped with a curved Ge(111) monochromator using Cu *K*α_1_ radiation (*λ* = 1.54056 Å). The scans covered the angular range from 5 to 90° 2θ. Rietveld refinement using the fundamental parameter approach was performed with *TOPAS* (Version 5; Coelho, 2018 ▸).

### Single-crystal diffraction   

Crystal data, data collection and structure refinement details are summarized in Table 1 ▸. Data for the modulated structure were integrated and corrected for Lorentz and polarization factors, before applying a numerical absorption correction with the program *JANA2006* (Petříček *et al.*, 2014 ▸). The structure was solved using the charge-flipping method of the program *SUPERFLIP* (Palatinus & Chapuis, 2007 ▸) implemented in the *JANA2006* software; the atomic positions were synchronized with those of the average structure. Structure refinement was performed with *JANA2006* against *F* ^2^ including anisotropic displacement parameters for all atoms. Second-order satellites were neglected because of their low intensity (about 99% of these reflections were found with intensities below 3σ) and two harmonic waves have been used for the fit of the atomic modulation functions.

### Scanning electron microscopy (SEM) and EDS   

SEM was performed with an SU8020 (Hitachi) with a triple-detector system for secondary and low-energy backscattered electrons (*U*_a_ = 5 kV). The composition of selected single crystals was determined by semiqu­anti­tative energy dispersive X-ray analysis (*U*_a_ = 20 kV) with a Silicon Drift Detector (SDD) X-Max^N^ (Oxford).

### Computational methods   

Solid-state calculations were performed with the all-electron code FHI-aims (Blum *et al.*, 2009 ▸) for three ordered structural models of LaTe_1.82(1)_. The FHI-aims calculations for subsequent real-space analysis were performed with a (2 × 2 × 2) *k*-point grid (model in *P*1) and a (3 × 2 × 2) *k*-point grid (model in *Amm*2 and *A*2) using the zeroth-order scalar relativistic zora correction, collinear spins, the numerical atom-centred basis of light level and the PBE functional (Perdew *et al.*, 1996 ▸). Real-space properties were evaluated subsequently with the help of the program package *DGrid* (Kohout, 2016 ▸). Hereby, the electron localizability indicator (ELI-D) was calculated on a grid with a 0.1 Bohr mesh size.

### Temperature-dependent electrical resistance   

The electrical resistance of LaTe_1.82(1)_ was measured between 7 and 345 K with a mini-CFMS (Cryogenic Ltd, London). Four gold contacts were attached to the surface of a single crystal in a linear set-up with a carbon conductive composite 7105 (DuPont) to establish the electrical contact between the crystal and the gold wires.

## Results and discussion   

### Synthesis   

Black plate-like single crystals of previously unreported LaTe_1.82(1)_ were obtained starting from the elements by alkali halide flux reactions or solid-state reactions with a small amount of I_2_ for mineralization in fused-silica ampoules. Temperatures above 1173 K in the presence of I_2_ lead to an attack on the ampoule wall, whereas temperatures of about 1073 to 1123 K are well suited for crystal growth, without noticeable side reactions with the ampoule material. The best results were achieved in a small gradient from 1123 to 1073 K, where crystals of about 0.3 mm in length were grown. The amount of added I_2_ needs to be compensated by excess La when preparing the experiment due to the formation of LaI_3_ during and after the experiment, which in turn alters the composition slightly. Solid LaI_3_ was mainly found at the sink of the ampoule, whereas the desired product was found at the source of the ampoule. A similar procedure at higher tem­per­atures was chosen, *e.g.* for *RE*Te_1.8_ (*RE* = Gd, Tb or Dy) (Poddig *et al.*, 2018 ▸).

### Diffraction image   

**Figure 1:**
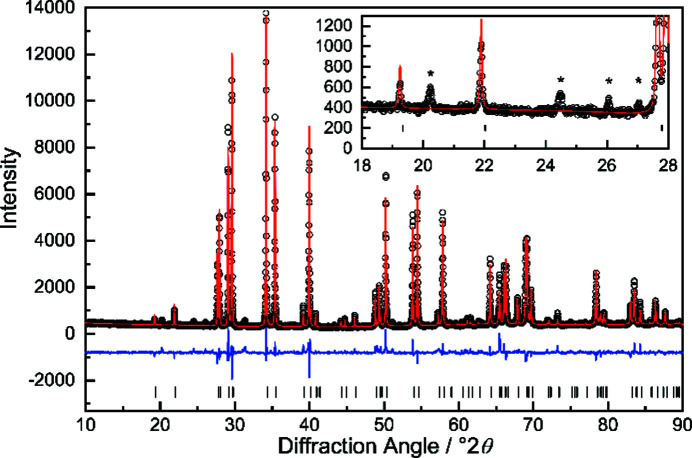
Rietveld refinement of LaTe_1.82(1)_. The space group of the aristotype ZrSSi (*P*4/*nmm*), with corresponding atom sites as the starting point for structure analyses, has been chosen. Satellite reflections are marked with an asterisk.

The strong reflections of the powder pattern can be indexed with a tetra­gonal unit cell with *a* = *b* ≃ 4.50 Å and *c* ≃ 9.17 Å. As a starting point for the Rietveld refinement, the space group (*P*4/*nmm*, No. 129) and the atom sites of the aristotype, the ZrSSi type, were chosen. The fit shows some additional unindexed reflections, which are not compatible with the space group *P*4/*nmm* and its most prominent lower symmetric subgroups (Fig. 1 ▸).

The diffraction image of a single crystal at ambient temperature reveals, as already indicated by powder diffraction, additional weak reflections in the layers (*hkn*) with *n* = 0, ±1, ±2,… (Fig. S1 in the supporting information). Slightly stronger reflections can be observed in the layers (*hkn*) with *n* = ±0.5, ±1.5,… (Fig. S1). These additional reflections can­not be indexed with a commensurate superstructure of the basic unit cell and were treated as satellites. Moreover, the distribution of the satellites with respect to the main reflections suggest a two-dimensional modulation. The whole diffraction image can then be indexed with five indices *hklm*_1_ *m*_2_ according to: 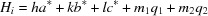 with 
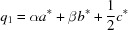
 and 
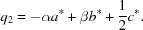



**Figure 2:**
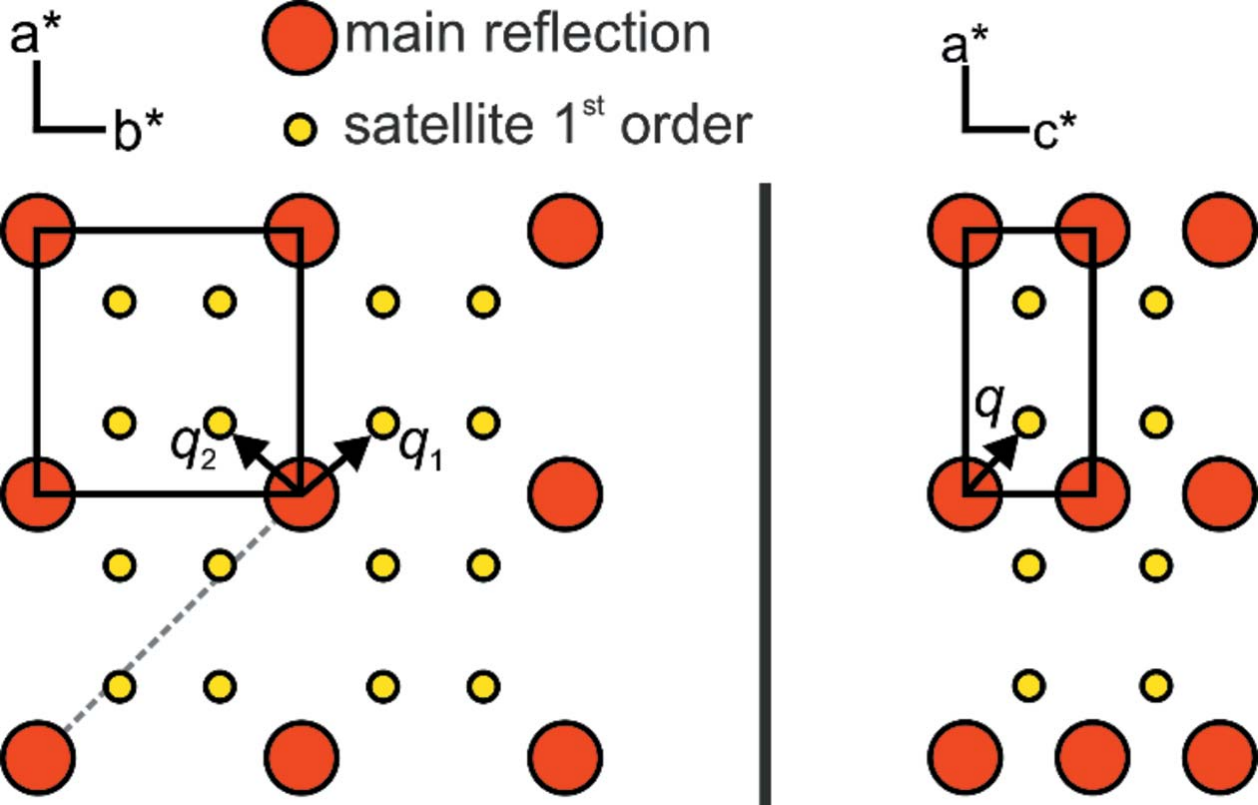
Projection of the relative position of the main reflections and first-order satellites along the [001] (left) and [010] (right) directions. The dotted line indicates the deviation of the satellite positions from the [110] mirror plane in Laue class 4/*mmm*.

The modulation wave vector components α and β were determined to be 0.272 (1) and 0.314 (1), respectively. A schematic image of the relative positions of the satellite reflections with respect to the main reflections is displayed in Fig. 2 ▸ and reconstructed precession images are shown in Fig. S1 (see supporting information). The schematic figure illustrates also that two different *q* vectors are necessary to index the complete diffraction image. Equivalent satellites are linked by a twofold rotational axis (Fig. S1), pointing towards ortho­rhom­bic (or lower) symmetry for the modulated structure. Furthermore, the weak additional reflections in the (*hkn*) (*n* = ±1, ±2,…) plane correspond to the linear combinations of *q*_1_ and *q*_2_, which cannot be explained by twinning and are, thus, evidencing a true [3 + 2]-dimensional modulated structure.

### Average crystal structure   

Single-crystal data collected at ambient temperature indicated the same lattice parameters for *a* and *b* within standard deviations so that a tetra­gonal unit cell of *a* = 4.4996 (5) and *c* = 9.179 (1) Å was chosen. The main reflections are clearly compatible with high tetra­gonal Laue symmetry and the space group *P*4/*nmm* (No. 129) was chosen for structure refinement of the average structure according to the reflection condition *h*+*k* = 2*n*. The refinement resulted in a reasonable structural model (Table S1 in the supporting information), with a reduced occupancy factor of the Te site in the Te layer of about 81.1 (4)%. The composition derived from semiqu­anti­tative EDS (energy-dispersive X-ray spectroscopy) measurements points towards a composition of LaTe_1.79 (1)_. Throughout the article, we will refer to this compound as LaTe_1.82(1)_, based on the refined composition of the modulated structure.

**Figure 3:**
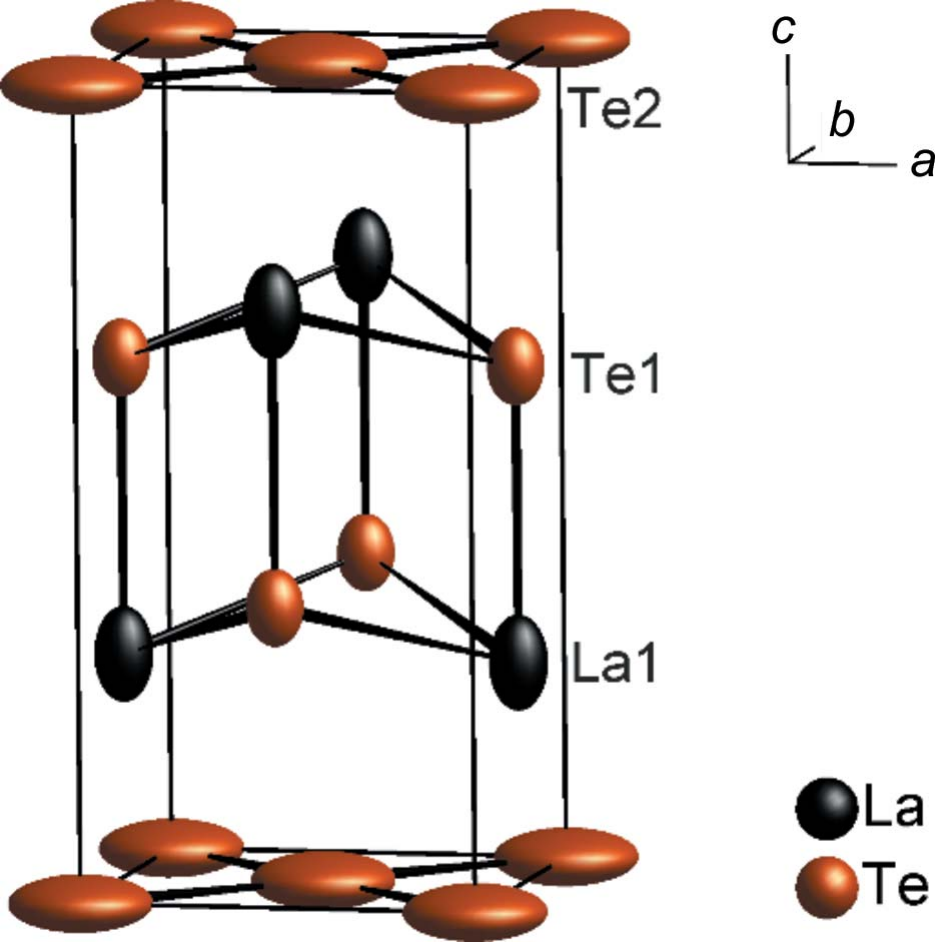
Average crystal structure of LaTe_1.82(1)_. Displacement ellipsoids are drawn with a probability level of 99.9%.

The average structure of LaTe_1.82(1)_ can be described with puckered [LaTe] layers sandwiched by square-planar [Te] layers. The partial occupation of the Te position in the [Te] layer, together with its large oblate anisotropic displacement parameters (ADPs) in the *ab* plane and prolate ADPs of the La atom along the [001] direction already give hints towards the modulation (Fig. 3 ▸).

The La atoms are regularly surrounded by five Te atoms of the puckered layer [4 × 3.3006 (4) and 1 × 3.324 (1) Å] and four Te atoms of the [Te] layer [4 × 3.3563 (7) Å], forming a regular tricapped trigonal prism. The planar [Te] layer is a perfect square net of Te atoms, with a Te—Te distance of 3.1821 (4) Å, which is significantly longer than a single Te—Te bond with about 2.80 Å [*e.g.* 2.78 Å in Rb_2_Te_2_, 2.86 Å in α-K_2_Te_2_ or 2.790 (1) Å in β-K_2_Te_2_; Böttcher *et al.*, 1993 ▸]. The large ADPs indicate a considerably reduced electron density and a positional shift of the Te atoms due to the formation of anionic Te units. Similar observations have been made for the incommensurable modulated selenides *RE*Se_1.85_ (*RE* = La–Nd or Sm) (Doert, Graf, Schmidt *et al.*, 2007 ▸; Graf & Doert, 2009 ▸).

### Refinement of the modulated structure   

**Figure 4:**
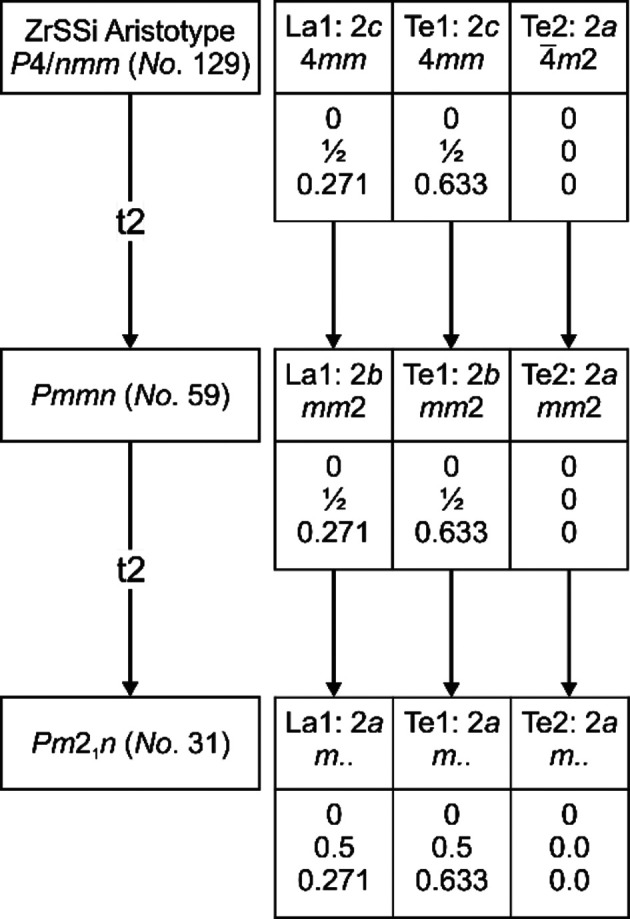
Group–subgroup relationship between the space group of the aristotpye ZrSSi (*P*4/*nmm*) and the chosen ortho­rhom­bic space groups of the basic structures.

The tetra­gonal symmetry of the average structure discussed above is violated by the modulation vectors, as mentioned before. The observed satellite positions are incompatible with a fourfold rotational axis (Fig. 2 ▸), resulting in a lower symmetric Laue class. To establish a suitable basic structure as starting model, ortho­rhom­bic and monoclinic subgroups of the space groups of the average structure were considered. The highest possible ortho­rhom­bic space group would then be *Pmmn*, which is a *translationengleiche* subgroup of the index 2 (t2) of *P*4/*nmm*, as displayed in Fig. 4 ▸. However, a very similar basic structure has been used for DySe_1.84_, with similar modulation wave vectors *q*_1_ = αa* + βb* + 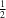 c* and *q*_2_ = αa* − βb* + 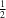 c*, with α = 0.333 and β = 0.273. The structure model obtained in superspace group *Pmmn*(α,β,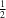)000(α,−β,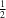)000 [No. 59.2.51.39 according to Stokes *et al.* (2011 ▸)] contained linear Se_3_^4−^ units besides Se_2_^2−^ and Se^2−^ anions. The existence of linear Se_3_^4−^ fragments, however, was ex­cluded due to ener­getic consideration based on the μ_2_-Hückel method (van der Lee *et al.*, 1997 ▸) and the final model for DySe_1.84_ was established in the noncentrosymmetric space group *Pm*2_1_ *n*(α,β,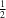)000(α,−β,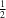)000 (No. 31.2.51.35). We there­fore decided to establish a second structure model for LaTe_1.82(1)_ in *Pm*2_1_ *n*(α,β,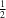)000(−α,β,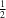)000, too, and evaluate it against the centrosymmetric one in *Pmmn*(α,β,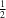)000(−α,β,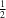)000. The group–subgroup relationship between these two space groups of the basic structures is displayed in Fig. 4 ▸, indicating a t2 group–subgroup relationship between them. The limits of both models shall be discussed in the following and the crystallographic details of the refinements are given in Table 1 ▸.

The main reflections meet the conditions for a primitive tetra­gonal lattice nearly perfectly, the data derived by powder diffraction and single-crystal diffraction give no hint of an ortho­rhom­bic or even monoclinic distortion of the lattice within standard uncertainties. However, taking the symmetry of the satellite reflections into account, the final unit-cell parameters were restrained to the conditions of an ortho­rhom­bic unit cell and were used for the integration of the intensities, as well as for structure refinements. According to the two modulation vectors and reflection conditions, the proper superspace group is *Pmmn*(α,β,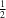)000(−α,β,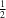)000 (No. 59.2.51.39). The modulated structure clearly contradicts twinning: the result of a fourfold rotational axis as twin element would result in four additional satellite reflections around the position of the main reflections in layers *hkl*, *l* = ±0.5, ±1.5,… (Fig. S2 in the supporting information). The reconstructed precession images, however, reveal only the four expected satellite reflections corresponding to ±*q*_1_ and ±*q*_2_.

A second model in superspace group *Pm*2_1_ *n*(α,β,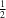)000(−α,β,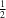)000 (No. 31.2.51.35; we keep the nonstandard setting for a concise structure description) has been evaluated against the centrosymmetric model to check if there are also differences in the structural model, as in the case of DySe_1.84_. For simplicity, we will use the three-dimensional space-group symbols during the structure descriptions to distinguish between the two different modulated structures in the following.

The refinement in *Pm*2_1_ *n* has been adjusted by considering inversion twinning. The refinement converged with a twin volume fraction of about 40% for the second domain.

**Figure 5:**
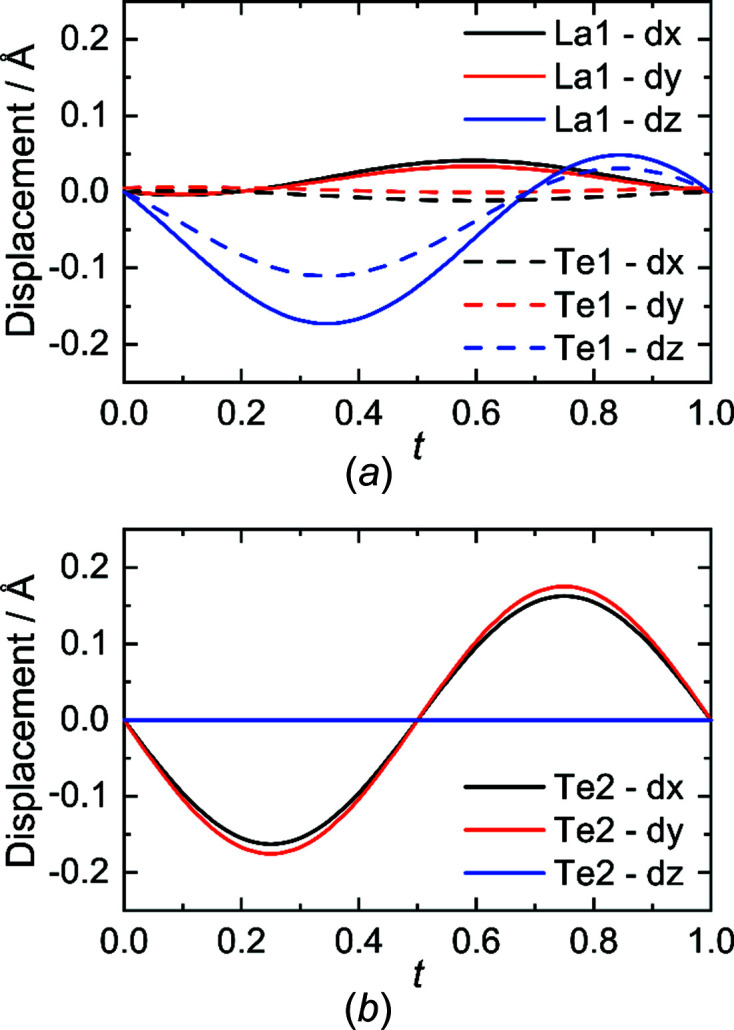
*t*-plots (*a*) of the displacement of La1 and Te1 in the [LaTe] layer and (*b*) of Te2 in the [Te] layer.

The atomic modulation functions (*amf*) were stepwise included in both refinements, by first modelling the positional displacement of all atoms, before including an additional occupational modulation for the Te2 atom. As already expected from the average structure, the La1 and Te1 position show mainly shifts in the *c* direction, whereas the Te2 atom in the [Te] layer shows a strong displacement in the *ab* plane, as displayed in the two *t* plots in Fig. 5 ▸. Note, that there is a small difference for the positional modulation along [001] for the Te2 atom between the models in *Pmmn* and *Pm*2_1_ *n*, as a result of the higher degree of freedom in the noncentrosymmetric space group. In *Pm*2_1_ *n*, the *t* plot shows a slightly sinusoidal curve in the *c* direction with a very small amplitude.

**Figure 6:**
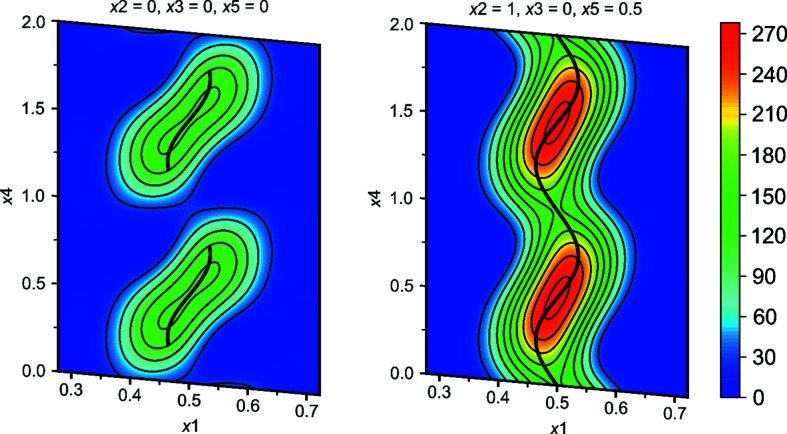
Fourier sections around Te2. The electron density is drawn in contour line steps of 30 e Å^−3^. The central thick black line indicates the refined modulation function.

The displacement in the [LaTe] layer along *c* can be explained as a reaction to the modulation in the [Te] layer; the La atom aims to compensate the missing Te atoms in the coordination sphere by getting closer to the [Te] layer. Consequently, the Te1 atom reacts accordingly to the La1 dislocation by adjusting its position along *c* as well. The displacement of the Te2 atom in the [Te] layer is slightly more pronounced (Fig. 5 ▸), due to vacancy formation and the creation of different Te anions. This holds for both models, as mentioned before. As a second step in the refinement, the occupational modulation in the [Te] layer was introduced by adding two harmonic functions. This improved the structural model in *Pmmn* and *Pm*2_1_ *n* considerably and the areas of low electron density at certain points in the Fourier map around Te2 are now also covered by the atomic modulation function (Fig. 6 ▸).

The Fourier sections in Fig. 6 ▸ also reveal a partially discontinuous behaviour of the electron density around Te2, although it is not very pronounced (see also Fig. S3 in the supporting information for a two-dimensional plot of the Te occupancy). The drawback in modelling this with harmonic functions are some overshooting and truncation effects in the final structure model, which we assume to be one major reason for the large residual electron-density maxima (see Table 1 ▸). The inter­atomic distances are, nevertheless, in good agreement with previously reported distances for Te anions and the refined composition matches that of the semiqu­anti­tative EDS analysis. In the second evaluated model in *Pm*2_1_ *n*, harmonic functions, as well as crenel functions, have been utilized. The refinement with harmonic waves in *Pm*2_1_ *n* converged with slightly better *R* values and a similar residual electron density compared to the refinement in *Pmmn*, mainly due to the greater number of independently refined parameters. The use of discontinuous functions, such as crenel or sawtooth functions (Petříček *et al.*, 2016 ▸), failed in *Pmmn* but refined stably in *Pm*2_1_ *n*, although they did not improve the structural model. The comparison between both types of functions suggests that treating the occupational and positional modulation of Te2 by harmonic functions is suitable. The large residual peaks in the difference Fourier (*F*_o_ – *F*_c_) maps decrease considerably if two modulation functions are applied to the ADPs of Te2 as well. However, this leads to nonpositive-definite values for *U*_min_ at some values of *t* and has hence been rejected for the final structure model.

### Discussion of the modulated crystal structure of LaTe_1.82(1)_   

The structure model derived in *Pmmn*(α,β,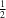)000(−α,β,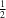)000 for LaTe_1.82(1)_ is used in the following paragraph for the discussion of the structural features as the structural differences between both models are negligible, as stated before.

The general feature of an alternating stacking of a puckered [LaTe] layer and [Te] layer is easily seen from Fig. S4 (see supporting information), which additionally shows the displacement of the La atoms along the *c* direction, as expected from the average structure. The motif of the puckered layer is very stable and does not show large deviations between different *REX*_2–*δ*_ compounds, whereas the planar [*X*] layer is the more inter­esting structural feature and will be discussed in the following.

**Figure 7:**
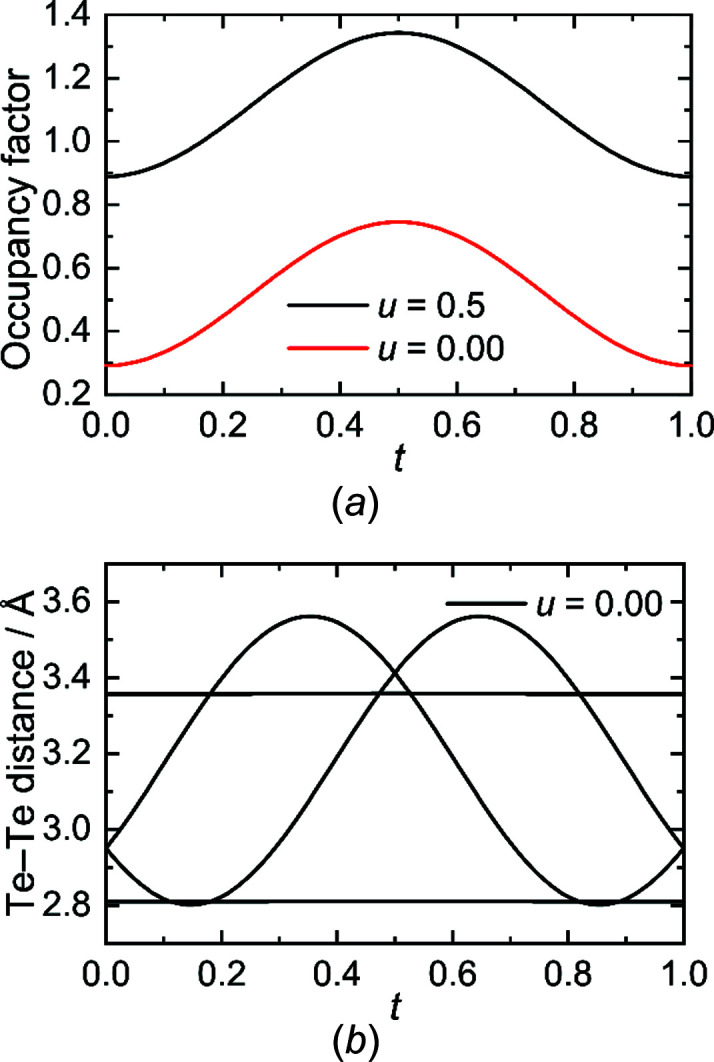
*t*-plots of (*a*) the occupation and (*b*) the distances shown for LaTe_1.82(1)_.

The change of the occupation and the variations of the Te—Te distances for the model in *Pmmn* are shown in Fig. 7 ▸. The *t*-plot for the occupational modulation displays a static behaviour along *t*, which is shifted for different *u* values. The changes of the Te—Te distances in the modulated [Te] layer are displayed in the second *t* plot (Fig. 7 ▸). Short distances with a lower limit of 2.801 (4) Å correspond to a Te—Te single bond (see above) and medium distances up to 2.959 (1) Å are in good agreement with distances reported for a linear Te_3_^4−^ anion (see, for example: Poddig *et al.*, 2018 ▸), as well as the often observed Te_2_^2−^ anions (see, for example: Stöwe, 2000*a* ▸). Larger distances of 2.984 (1) to 3.563 (4) Å are mainly considered as nonbonding inter­actions between Te entities. Compared to the known rare earth metal polysulfides and selenides, the inter­pretation of the Te—Te distances from a purely crystallographic viewpoint is more difficult as we face a much larger variety of distances, and boundaries between bonding and nonbonding inter­actions in the *RE*Te_2–δ_ system are floating. Reported Te—Te distances in dinuclear Te_2_^2−^ anions ranging from 2.868 (1) Å in GdTe_1.8_ (Poddig *et al.*, 2018 ▸) to 3.036 (2) Å in LaTe_2_ (Stöwe, 2000*a* ▸) have been inter­preted as single bonds. Nevertheless, the observed distances in the modulated structure of LaTe_1.82(1)_ are in good agreement with the distances found in comparable com­pounds.

**Figure 8:**
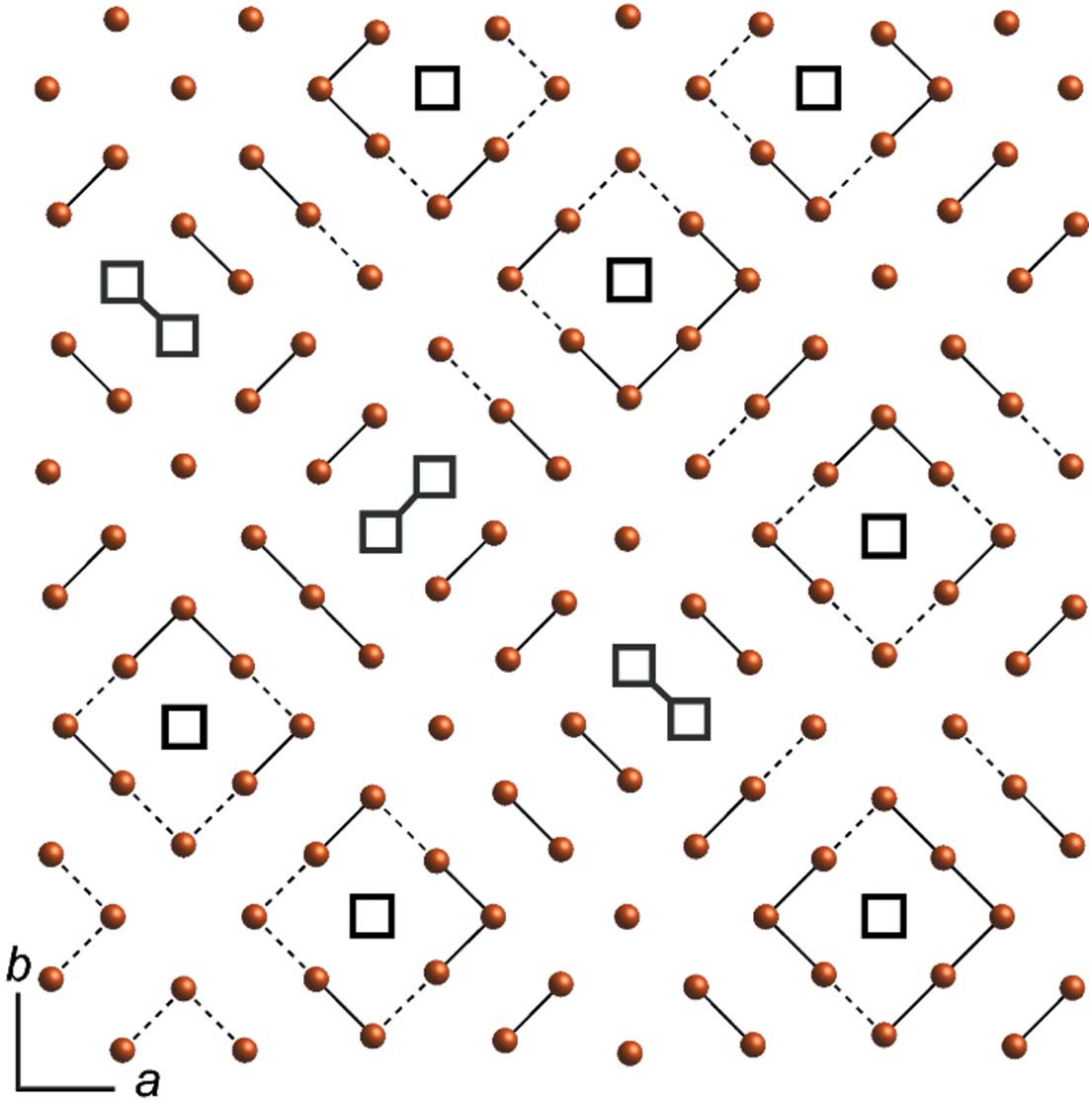
Section of the modulated Te layer, with a cut-off occupancy at 0.5. Black-framed squares emphasize the voids in the layer. Solid lines are drawn from 2.801 to 2.981 Å and dashed lines are drawn for distances between 2.984 to 3.282 Å.

A section of the modulated [Te] layer is depicted in Fig. 8 ▸, with Te positions displayed for a refined occupancy factor of 0.5 and greater. Solid lines indicate distances from 2.801 (4) up to 2.981 (2) Å, which should illustrate probable bonding situations; dashed lines are drawn up to 3.282 (2) Å for a better visualization of possible larger fragments, which have been reported for other polychalcogenides. Considering only these distances, three different motifs can be distinguished: a Te_8_ unit, probably consisting of smaller anions, like Te^2−^, Te_2_^2−^, bent Te_3_^2−^ and linear Te_3_^4−^ anions, as well as isolated Te^2−^ anions surrounded by different anionic Te entities. Eight-membered rings of chalcogen atoms and isolated *X* ^2−^ anions are, as already pointed out, common motifs in the crystal structures of the rare earth metal sulfides and selenides with compositions of *RE*S_1.9_ (*RE* = La–Nd, Sm, Gd–Tm) (Doert, Graf, Lauxmann *et al.*, 2007 ▸; Tamazyan *et al.*, 2000 ▸; Müller *et al.*, 2012 ▸), *RE*Se_1.9_ (*RE* = La–Nd, Sm, Gd–Tm) (Grupe & Urland, 1991 ▸; Plambeck-Fischer *et al.*, 1989 ▸; Urland *et al.*, 1989 ▸; Müller *et al.*, 2012 ▸), *RE*_8_S_15–δ_ (*RE* = Y, Tb–Ho) (Doert *et al.*, 2012 ▸), *RE*_8_Se_15–δ_ (*RE* = Y, Gd–Er; δ = 0 ≤ δ ≤ 0.3) (Doert, Dashjav *et al.*, 2007 ▸) and *RE*Se_1.85_ (*RE* = La–Nd or Sm) (Doert, Graf, Schmidt *et al.*, 2007 ▸; Graf & Doert, 2009 ▸).

In the modulated [Te] layer of LaTe_1.82(1)_, a Te_4_ square is apparent additionally when choosing small cut-off values for the approximant crystal structure for visualization (see, for example: Fig. S5 in the supporting information). As all four atoms in these fragments have an occupation value of about 0.52 (5), the presence of all four at the same time seems unrealistic. Instead, an unresolved superposition of a dinu­clear Te_2_^2−^ anion with two adjacent vacancies is the most likely explanation. Moreover, there is no evidence for anionic [Te_4_] squares with Te—Te distances of 2.80 Å in the literature. Cationic Te_4_^2+^ and the corresponding Se_4_^2+^ entities, on the other hand, are well known (see, for example: Barr *et al.*, 1968 ▸; Beck *et al.*, 1997 ▸; Forge & Beck, 2018 ▸; Ruck & Locherer, 2015 ▸) and their bonding situation has been investigated by computational methods in 1980 already (Rothman *et al.*, 1980 ▸). The typical Te—Te distance in Te_4_^2+^ is about 2.65 to 2.70 Å (Ruck & Locherer, 2015 ▸) and the Te—Te—Te angles are often close to 90°, which results in a slight distortion from idealized *D*_4*h*_ symmetry. Taking the EDS results and the site-occupation factor from the average structure (both resulting in a composition of about LaTe_1.8_) into account, a substantial number of voids in the [Te] layer is expected, which also supports the idea of a disordered motif of dinuclear Te_2_^2−^ anions and adjacent vacancies instead.

A similar discussion of apparent structure motifs and possible (super)positions due to unresolved disorder shall be deduced for the apparent Te_8_ rings (Fig. 8 ▸). Arrangements of disordered *X*_2_^2−^ anions have been identified as the constituents of eight-membered ring-like motifs in the sulfides *RE*_8_S_15–δ_ (*RE* = Y, Tb–Ho; Doert *et al.*, 2012 ▸) and selenides *RE*_8_Se_15–δ_ (*RE* = Y, Gd–Er; δ = 0 ≤ δ ≤ 0.3; Doert, Dashjav *et al.*, 2007 ▸). In LaTe_1.82(1)_, these apparent eight-membered rings may also consist of different disordered constituents, like Te_3_^2−^ and Te_3_^4−^ anions, along with the more common Te^2−^ and Te_2_^2−^ motifs, around central vacancies. However, this is hard to resolve solely from the diffraction data. To gain more insight into this structural motif and the chemical bonding situation in the modulated [Te] layers in general, chemical bonding analyses were performed for three different com­mensurate approximants.

### Bonding analysis   

Quantum mechanical calculations based on density functional theory (DFT) and bonding analyses with the electron localizability indicator (ELI-D) (Kohout, 2004 ▸, 2006 ▸; Pendás *et al.*, 2012 ▸) have been performed for three approximant structures of LaTe_1.82(1)_ in order to provide additional information on the bonding situation in the Te layers, especially regarding the (presumably disordered) Te_4_ and Te_8_ entities. As these calculations require three-dimensional commensurate structure models as bases, a suitable commensurate ortho­rhom­bic *B*-centred 4 × 3 × 2 supercell of the basic ZrSSi-type structure was chosen by approximating the *q* vector components α and β by 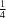 and 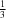, respectively. The respective three-dimensional space groups and the atomic positions were derived by the *JANA2006* software package (Petříček *et al.*, 2014 ▸) by enabling the commensurate option, after the final refinement of the modulated structure. According to the previously reported structures of *RE*Se_1.875–δ_ compounds (Doert, Dashjav *et al.*, 2007 ▸; Stöwe, 2001 ▸), this cell was transformed into an *A*-centred setting for a better comparison, resulting in a 3 × 4 × 2 supercell with unit-cell dimensions of *a* = 13.4859 (4), *b* = 17.9812 (4) and *c* = 18.3446 (8) Å. As the highest possible symmetry, space group *Amm*2 (No. 38), the space group of the *RE*_8_Se_15–δ_ compounds, was chosen for one approximant. The respective *Amm*2 structure model exhibits bent Te_3_ units in the Te_8_ ring, as well as a Te_4_ square, which cannot be resolved due to the symmetry restrictions in this space group (Fig. S6 in the supporting information). A second model has been established in the space group *A*2 (No. 5), *i.e.* removing the two perpendicular mirror planes. This space group has also been used to describe the disorder in the structures of the compounds *RE*_8_S_15–δ_. Here, only Te_2_^2−^ anions with alternating short (bonding) and longer (nonbonding) distances are considered as building units of the Te_8_ rings (Fig. S7 in the sup­porting information), enabling a direct comparison between the bent *X*_3_ fragments in *Amm*2, and the *X*_2_^2−^ patterns known from different *REX*_1.9_ and *RE*_8_ *X*_15–δ_ structures (*cf*. above). A third model in the space group *P*1 (No. 1) was developed starting from the previous model in *A*2 to lift the symmetry restrictions completely. The disorder of the apparent Te_4_ unit can then be resolved by assuming two vacancies and a single Te_2_^2−^ anion (Fig. S8 in the supporting information). Bear in mind that energetic comparisons are only possible between models with the same number of atoms. This is the case for the models in *Amm*2 and *A*2, but not for *P*1, due to the additional vacancies when taking the occupational disorder of the Te_4_ square into account. This means that an identification of the favoured structure is not possible based on the computed net energies only.

The calculated band gaps for all models are finite, but small, *e.g.* 0.04 eV for the model in *A*2, so that semiconducting electronic properties are expected (see below). The corresponding stoichiometric LaTe_2_ was reported as metallic (Stöwe, 2000*a* ▸).

Regarding the large Te_8_ entities, the (disordered) structure model in *Amm*2 shows a lower energy than the corresponding (ordered) model in *A*2 (Δ*E* = 0.16 eV). The *Amm*2 structure would imply the existence of bent Te_3_ entities with bond angles of about 90.0 (1)° in the planar Te layer, as mentioned before. Angular Te_3_^2−^ anions are well known, *e.g.* from the binary dialkali metal tritellurides *A*_2_Te_3_ with *A* = K, Rb or Cs (Eisenmann & Schäfer, 1978 ▸; Böttcher, 1980 ▸) and have Te—Te distances of about 2.80 Å, but significantly larger bond angles of about 100°. These Te_3_^2−^ anions are, however, more or less isolated in the structures and no inter­actions amongst them or with other anionic fragments are expected. Bent Te_3_ entities with Te—Te—Te angles close to 90° were described as parts of the anionic substructure in disordered polytellurides like K*RE*_3_Te_8_ (Stöwe *et al.*, 2003 ▸; Patschke *et al.*, 1998 ▸) and RbUSb_0.33_Te_6_ (Choi & Kanatzidis, 2001 ▸), and in the modulated structures of K_1/3_Ba_2/3_AgTe_2_ (Gourdon *et al.*, 2000 ▸), LnTe_3_ (Malliakas *et al.*, 2005 ▸) and *RE*SeTe_2_ (Fokwa *et al.*, 2002 ▸; Fokwa Tsinde & Doert, 2005 ▸), for example.

**Figure 9:**
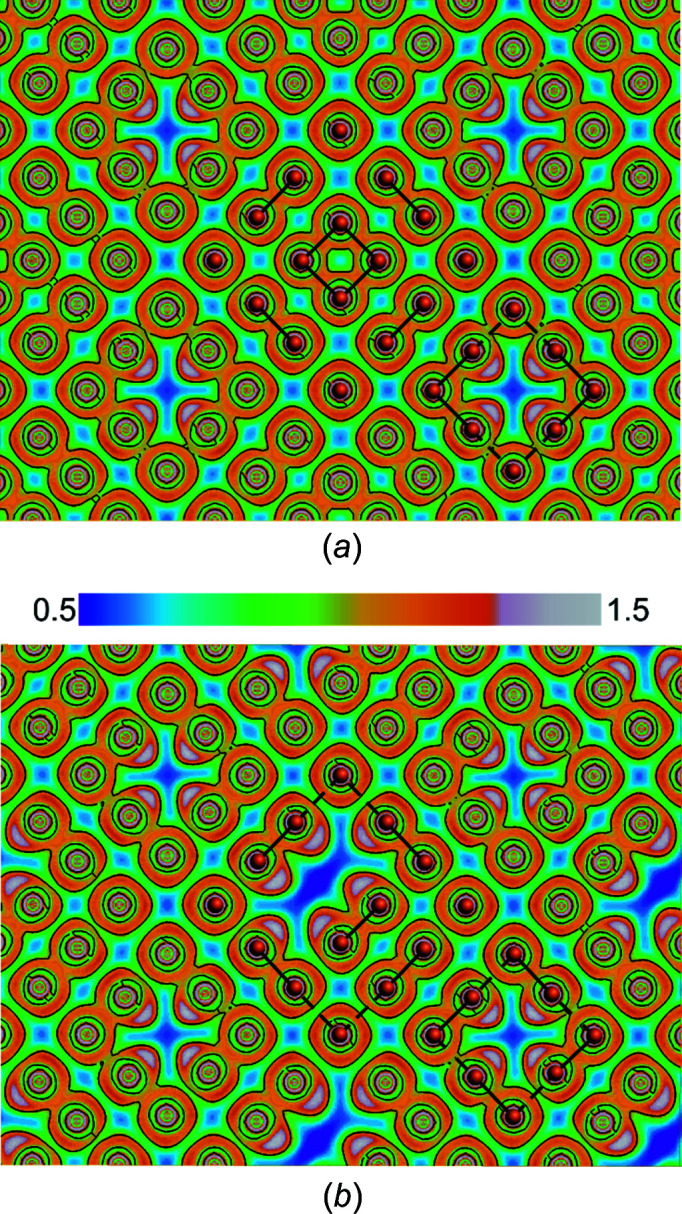
Orthoslices of ELI-D of the Te layer of LaTe_1.82(1)_ with isocontour lines. (*a*) The largely unordered model in *Amm*2, with bent Te_3_ units in the Te_8_ entities and a Te_4_ square. (*b*) The essentially ordered model in *P*1, exhibiting linear Te_2_^2−^ units in the Te_8_ entities and with only one Te_2_^2−^ anion instead of a Te_4_ square. Both pictures hint towards the inter­actions between different Te fragments in the planar layer.

Orthoslices of the ELI-D within the [Te] layers of the *Amm*2 and the *P*1 approximant are shown in Fig. 9 ▸. The isolines of both ELI-D images within the [Te] plane discriminates most of the observed Te atoms in three groups: isolated Te^2−^, dinuclear Te_2_^2−^ and bent trinuclear Te_3_^2−^ anions. The ELI-D in the *P*1 model suggest a slightly more pronounced tendency to form Te_2_^2−^ dumbbells as main polynuclear building units, in accordance with the reported structures of rare earth metal sulfides and selenides (Doert & Müller, 2016 ▸) and with theoretical considerations for the rare earth metal selenides (Lee & Foran, 1994 ▸). As discussed above, these entities are expected to represent the dominant bonding inter­actions in the planar layer, but the local bonding situation and the stability of the corresponding fragment are also influenced by inter­actions with other telluride anions in the [Te] layers, as well as by the surrounding La atoms in the layers below and above. Indeed, substantial inter­actions between these small anionic fragments in the [Te] layer have to be considered based on relatively high isovalues of the ELI-D between the dominating species in all models (Fig. 9 ▸). For nonbonding or anti­bonding inter­actions, deep valleys (depicted in blue) would be expected, like, for example, the dark-blue regions between strongly localized lone-pair regions in *P*1.

The ELI-D slices in Fig. 9 ▸ show some additional inter­esting features. Significant localized lone-pair regions are found for those Te atoms located directly adjacent to vacancies. This may be taken as evidence for the anionic character of the [Te] layers. The respective lone pairs are localized in the structural voids with no hint of bonding inter­actions between Te fragments encasing the voids. The additional vacancies of the *P*1 model (Fig. 9 ▸ *b*) seem to be used to accommodate the lone pairs of different anionic Te fragments, again supporting the ionic description of the [Te] layer and in accordance with the calculated band gap and the measured semiconducting properties of LaTe_1.81 (2)_ (see below).

Note, that the evaluated approximant structures indicate compositions of about LaTe_1.95_ (*Amm*2 and *A*2) and LaTe_1.875_ (*P*1), *i.e.* a higher tellurium content as compared to the actual composition LaTe_1.82(1)_. Thus, additional vacancies would be necessary to get a more realistic image of the Te substructure. The bonding features between different constituents should nevertheless be comparable.

### Electrical resistance of LaTe_1.82(1)_   

**Figure 10:**
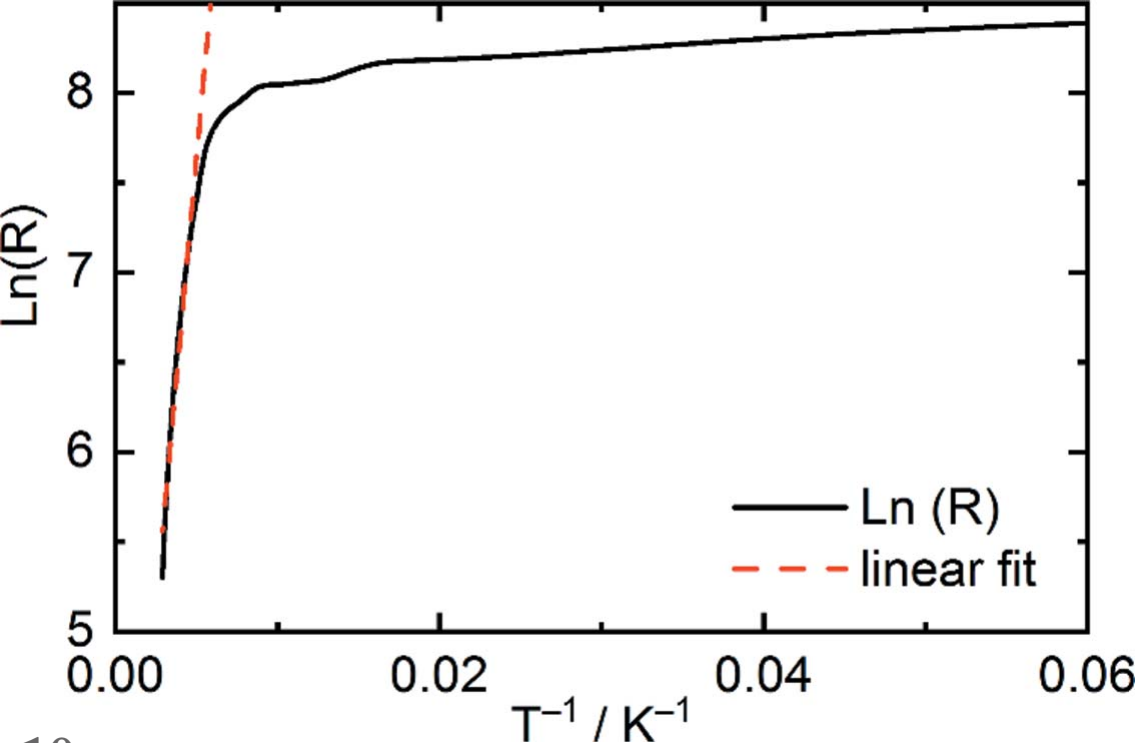
Temperature-dependent electrical resistance of LaTe_1.82(1)_ between 7 and 345 K, showing a semiconducting behaviour.

The temperature-dependent electrical resistance of LaTe_1.82(1)_ has been recorded by a four-point measurement between 7 and 345 K. The observed temperature dependence of the resistance of LaTe_1.82(1)_ is characteristic for a semiconductor (Fig. 10 ▸). The band gap, *E*_g_, can be estimated from the highest temperature ρ values using a fit of the form ρ = ρ_0_exp(*E*_g_/2*k*_B_ *T*), where *k*_B_ is the Boltzmann constant and *T* is the absolute temperature. The estimated *E*_g_ value is 0.17 eV for LaTe_1.82(1)_, which is slightly larger than the calculated value for the model in *A*2 (*cf*. above). However, comparable com­pounds like NdTe_1.89 (1)_, GdTe_1.8_ and SmTe_1.84_ show similar band gaps of 0.14 (Stöwe, 2001 ▸), 0.19 (Poddig *et al.*, 2018 ▸) and 0.04 eV (Park *et al.*, 1998 ▸), respectively, in contrast to LaTe_2_, which was reported to be metallic (Stöwe, 2000*a* ▸).

## Conclusions   

The modulated structure of the rare earth metal polytelluride LaTe_1.82(1)_ has been solved and refined using the superspace approach. The diffraction pattern evidences that the tetra­gonal symmetry of the average structure is not preserved in the modulation. Two different models evaluated in superspace groups *Pmmn*(α,β,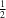)000(−α,β,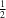)000 (No. 59.2.51.39) and *Pm*2_1_ *n*(α,β,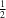)000(−α,β,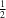)000 (No. 31.2.51.35) show only slightly different results, suggesting that the highest possible superspace group *Pmmn*(α,β,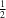)000(−α,β,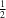)000 should describe the structure accordingly. In the regime of the different *REX*_2–δ_ compounds, LaTe_1.82(1)_ may be best described as a depleted *REX*_1.9_ or *REX*_1.875_ structure. For the latter two structure types, it is possible to accommodate the respective anionic vacancies structurally isolated, *i.e.* separated between different (poly)telluride anions and in a commensurate superstructure. In the title compound, 18% of the Te2 positions are unoccupied, which leads to two obvious consequences: a commensurate ordering of vacancies and remaining anions is not possible anymore, and a considerable number of adjacent anion defects occur. In other words, LaTe_1.82(1)_ exhibits a higher propensity for missing Te_2_^2−^ dianions. This description deviates significantly from the structures described for *RE*Te_1.8_ (*RE* = Sm, Gd–Dy; Ijjaali & Ibers, 2006 ▸; Wu *et al.*, 2002 ▸; Gulay *et al.*, 2007 ▸; Poddig *et al.*, 2018 ▸). However, this different structure fits well with the overall trend for the rare earth metal tellurides *RE*Te_2–δ_, where different structures have been observed for a similar composition, as pointed out for the stoichiometric *RE*Te_2_ (*RE* = La, Ce or Pr) compounds. Quantum mechanical calculations based on DFT with subsequent ELI-D-based bonding analysis for the ionic Te layer reveal Te_2_^2−^ units as dominant species, however, with significant long-range inter­actions amongst them. Temperature-dependent resistance measurements suggest a semiconducting behaviour with a band gap of about 0.17 eV, which is in good agreement with comparable rare earth metal compounds.

## Supplementary Material

Crystal structure: contains datablock(s) global, I, II. DOI: 10.1107/S2053229620005094/sk3745sup1.cif

Structure factors: contains datablock(s) I. DOI: 10.1107/S2053229620005094/sk3745Isup2.hkl

Additional figures and tables. DOI: 10.1107/S2053229620005094/sk3745sup3.pdf

CCDC references: 1982148, 1996576

## Figures and Tables

**Table 1:** Crystallographic data and refinement details for LaTe_1.82(1)_

	Model *Pmmn*				Model *Pm*2_1_ *n*			
Refined composition	LaTe_1.811 (4)_				LaTe_1.825 (3)_			
Formula weight (g mol^−1^); *F*(000)	370.8; 303				371.8; 304			
Crystal size (mm^−3^)	0.0588 × 0.0472 × 0.0094							
Diffractometer, radiation	Bruker Kappa APEX II, Mo *K*α (0.71073 Å)							
Temperature	296 (1) K							
Lattice parameters (Å)	*a* = 4.5020 (5), *b* = 4.4985 (5), *c* = 9.181 (1)							
	α = β = γ = 90°							
Modulation vectors	*q*_1_ = αa* + βb* + γc*							
	*q*_2_ = −αa* + βb* + γc*							
	α = 0.272 (1), β = 0.314 (1), γ = 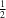							
Index range measured	−7≤*h*≤8; −8≤*k*≤8; −16≤*l*≤9; −1≤*m*,*n*≤1							
	2.18≤θ≤38.36							
Measured reflections	30619							
Absorption coefficient μ (mm)	25.201							
*T*_min_, *T*_max_	0.3929, 0.4983							
Extinction parameter (Becker & Coppens, 1974 ▸)	0.151				0.153			
Independent reflections	1757, 738 > 3σ(*I*)				6103, 1386 > 3σ(*I*)			
Main reflection	415, 323 > 3σ(*I*)				1080, 719 > 3σ(*I*)			
First-order satellites	1342, 415 > 3σ(*I*)				3946, 664 > 3σ(*I*)			
*R*_int_; *R*_σ_	0.0556, 0.0461 for *I* > 3σ(*I*)				0.0469, 0.0600 for *I* > 3σ(*I*)							
	0.1563, 0.1934 for all				0.1435, 0.2810 for all			
Superspace group, *Z*	*Pmmn*(α,β, 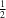 )000(−α,β, 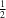 )000 (No. 59.2.51.39), 2				*Pm*2_1_ *n*(α,β, 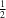 )000(−α,β, 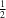 )000 (No. 31.2.51.35), 2			
Refinement method	*JANA2006*, full-matrix against *F* ^2^							
Restrictions/parameters	0/33				0/66			
	*R*_1_ [3σ(*I*)]	*R*_1_ (all)	*wR*_2_ [3σ(*I*)]	*wR*_2_ (all)	*R*_1_ [3σ(*I*)]	*R*_1_ (all)	*wR*_2_ [3σ(*I*)]	*wR*_2_ (all)
All reflections	0.0495	0.1300	0.0872	0.1111	0.0506	0.2131	0.0883	0.1287
Main reflections	0.0247	0.0385	0.0509	0.0542	0.0326	0.0554	0.0575	0.0610
First-order satellites	0.1079	0.2470	0.1891	0.2479	0.1000	0.3333	0.1949	0.2928
Goodness-of-fit [3σ(*I*)/all]	1.53/1.26				1.30/0.90			
Largest difference peak/hole (e Å^−3^)	15.11/−17.25				13.21/−14.87			

Computer programs: *APEX2* (Bruker, 2010 ▸), *SAINT-Plus* (Bruker, 2017 ▸), *SHELXT2014* (Sheldrick, 2015*a* ▸), *SUPERFLIP* (Palatinus & Chapuis, 2007 ▸), *SHELXL2019* (Sheldrick, 2015*b* ▸), *JANA2006* (Petříček *et al.*, 2014 ▸), *OLEX2* (Dolomanov *et al.*, 2009 ▸), *SADABS* (Krause *et al.*, 2015 ▸) and *DIAMOND* (Brandenburg, 2019 ▸).
