# Supplementary material for: LaTe1.82(1): modulated crystal structure and chemical bonding of a chalcogen-deficient rare earth metal polytelluride
Source: Acta Crystallogr C Struct Chem. 2020 May 6;76(Pt 6):530–40. doi: 10.1107/S2053229620005094 (PMC7273186; doi:10.1107/S2053229620005094)
Supplement: Supplementary file 3 [file c-76-00530-sup3.pdf]

STRUCTURAL CHEMISTRY

Volume 76 (2020)

Supporting information for article:

LaTe1.82(1): modulated crystal structure and chemical bonding of a chalcogen-deficient rare earth metal polytelluride

Dedicated to Professor Stephen Lee on the occasion of his 65th birthday.

Hagen Poddig, Kathi Finzel and Thomas Doert

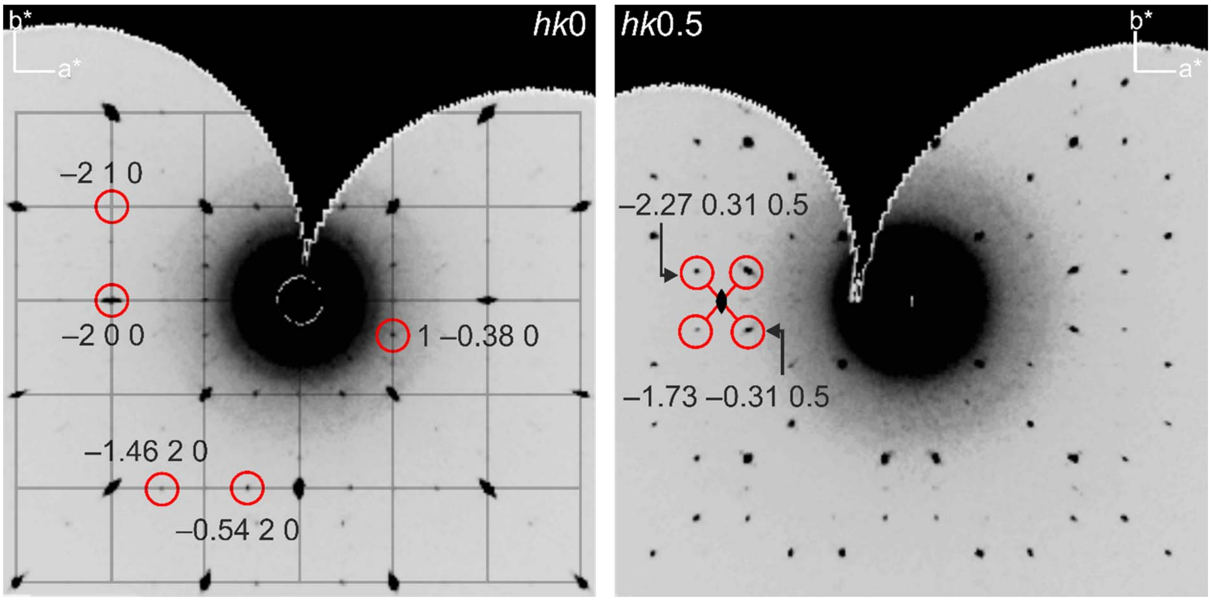

Figure S1 Reconstructed precession image of the planes *hk*0 and *hk*0.5. Miller indices correspond to the basic tetragonal cell with $a = b \approx 4.50$ Å and $c \approx 9.17$ Å (grid on left image). The *hk*0 plane shows the main reflections, which are only observed for $h + k = 2n$, and the cross-term satellite reflections. First order satellites in the *hk*0.5 plane demonstrate the wave vector component of ½ along *c** and the incompatibility with a fourfold rotational axis.

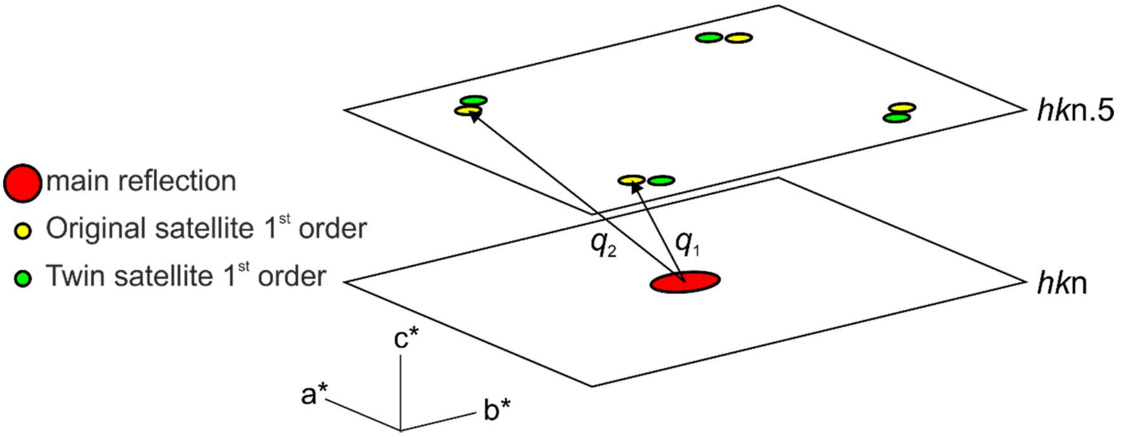

Figure S2 Sketch simulating reflections in the *hk*n.5 layers, in case of twinning (fourfold rotation axis as tin law).

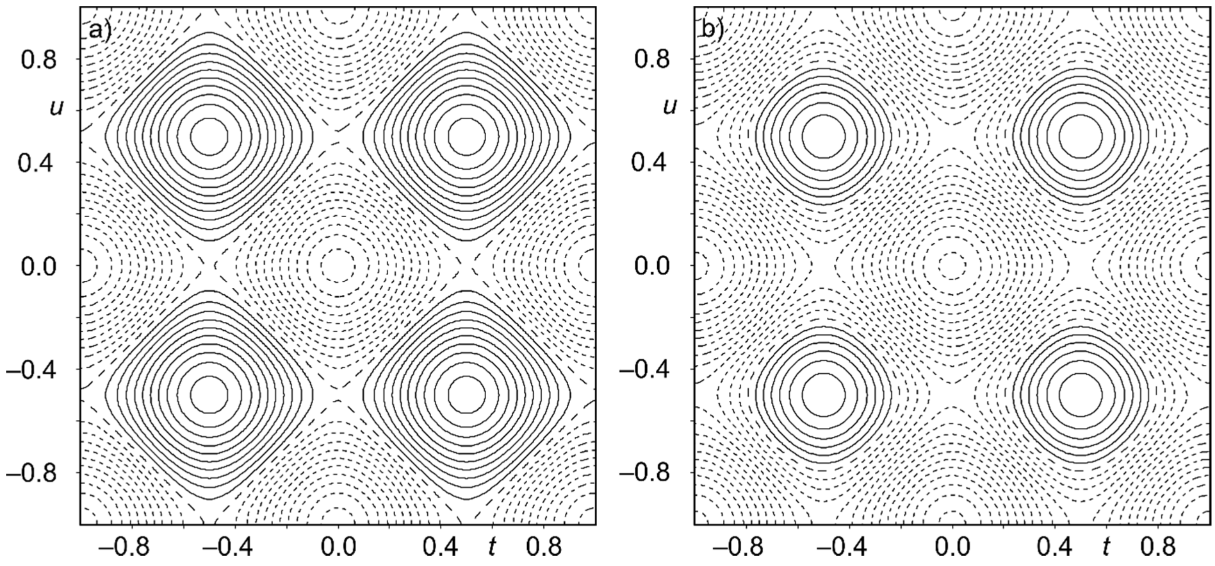

Figure S3 2D *t*-Plots of $LaTe_{1.82(1)}$. a) Position of Te2 in *dz* depending on *t* and *u*. b) Occupancy of Te2 depending on *t* and *u* with a reference level of 0.5. Both diagrams indicate a very similar behaviour along both modulation directions.

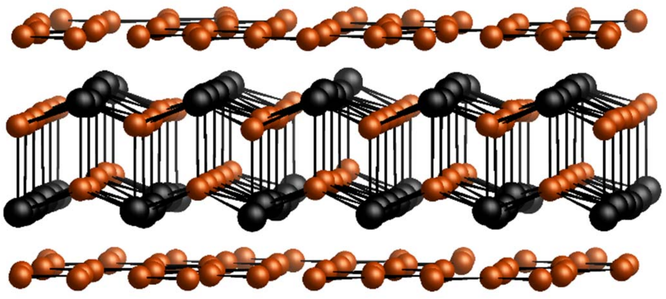

Figure S4 Approximate structure of $LaTe_{1.82(1)}$, displaying the effect of the modulation on the puckered [LaTe] layer.

Table S1 Crystallographic data and refinement details for the average structure of $LaTe_{1.82(1)}$.

Crystal data	
Chemical formula	$LaTe_{1.81(1)}$
M_r	370.50
Crystal system, space group	Tetragonal, *P*4/*nmm*
Temperature (K)	296(1)
a, *c* (Å)	4.4996 (5), 9.179 (1)
Z	2
Radiation type	Mo *K*α
μ (mm^{-1})	25.18
Crystal size (mm)	0.03 × 0.02 × 0.02

Data collection	
Diffractometer	Bruker *APEX*-II CCD
Absorption correction	multi-scan, *SADBS,* (Krause *et al.*, 2015)
T_{min}, T_{max}	0.641, 0.748
No. of measured, independent and observed [$I > 2\sigma(I)$] reflections	3402, 351, 289
R_{int}	0.046
θ values (°)	θ_{max} = 38.5, θ_{min} = 2.2
$(\sin \theta/\lambda)_{max}$ (Å^{-1})	0.875
Refinement	
$R[F^2 > 2\sigma(F^2)]$, $wR(F^2)$, S	0.024, 0.057, 1.07
No. of reflections	351
No. of parameters	11
$\Delta\rho_{max}$, $\Delta\rho_{min}$ (e×Å^{-3})	3.15, −2.35

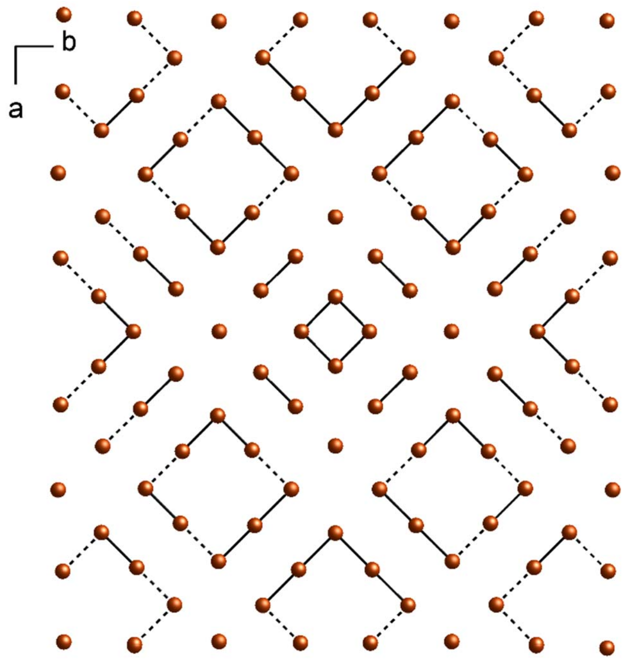

Figure S5 Section of the modulated Te layer with a cut-off occupancy at 0.45. The Te_4 artifact as result of the refinement is displayed in the center of the image.

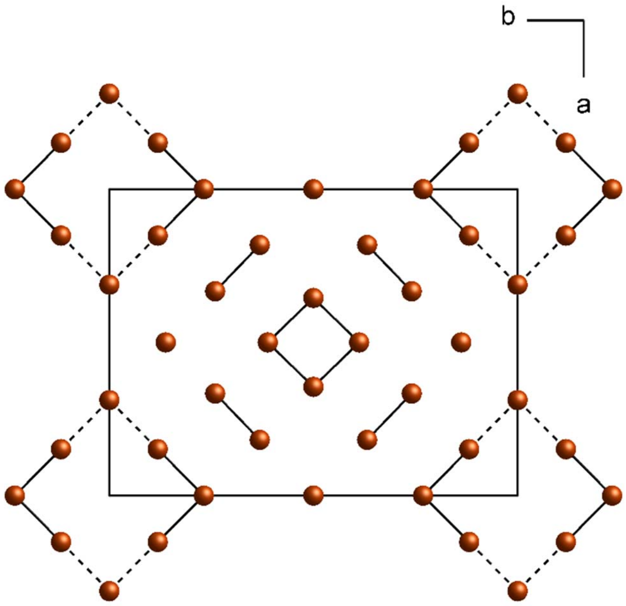

Figure S6 Structure approximant in *Amm*2 with bent Te_3 unit in the Te_8 ring and an unresolved disorder of Te_2^{2-} anions in the quadratic Te_4 fragment.

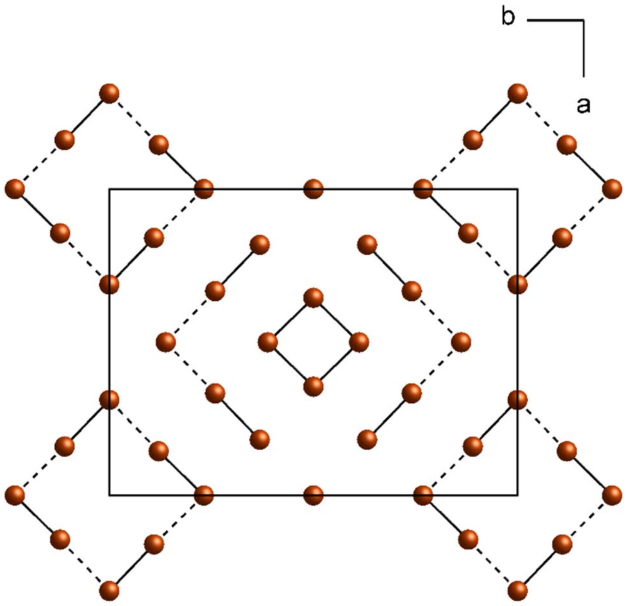

Figure S7 Structure approximant in *A*2, only showing Te_2^{2-} anions as fragments of the Te_8 ring and an unresolved disorder of Te_2^{2-} anions in the quadratic Te_4 fragment.

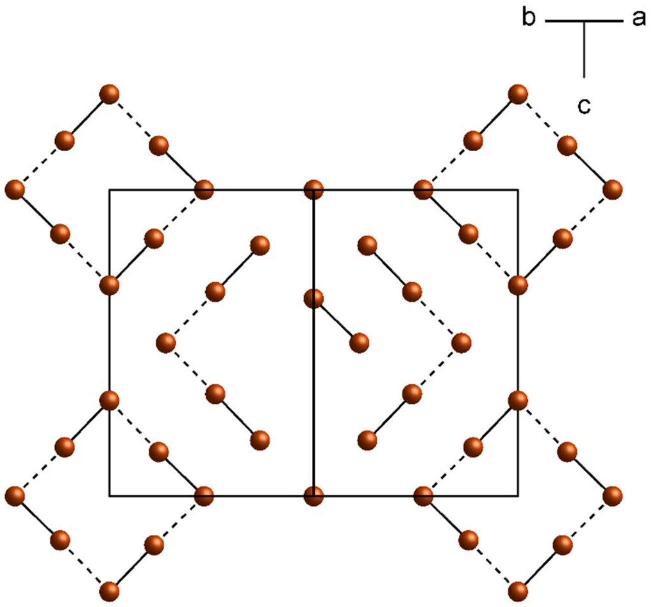

Figure S8 Structure approximant in *P*1, only showing Te_2^{2-} anions as fragments of the Te_8 ring and the resolved disorder of Te_2^{2-} anions in the center.
